# Preditores de Readmissão Hospitalar Após Cirurgia de Revascularização Miocárdica – Reflexões e Perspectivas

**DOI:** 10.36660/abc.20250133

**Published:** 2025-04-07

**Authors:** Aurora Felice Castro Issa

**Affiliations:** 1 Instituto Nacional de Cardiologia Rio de Janeiro RJ Brasil Instituto Nacional de Cardiologia, Rio de Janeiro, RJ – Brasil; 2 IDOMED Rio de Janeiro RJ Brasil IDOMED, Rio de Janeiro, RJ - Brasil

**Keywords:** Readmissão do Paciente, Cirurgia Geral, Revascularização Miocárdica

Aspectos como o alto custo do cuidado em saúde, a sobrecarga dos profissionais de saúde e o envelhecimento populacional com consequente aumento das doenças crônicas evidenciam a necessidade de atenção à qualidade na assistência cardiovascular de alta complexidade, especialmente na cirurgia cardíaca.^1^ Nesse cenário, os indicadores de qualidade apresentam-se como instrumento de extrema importância na avaliação assistencial.

Vários são os indicadores de qualidade atualmente disponíveis e um deles é a taxa de readmissão hospitalar que pode ser vista como um desfecho muitas vezes utilizado como secundário e apesar de ser bastante promissor, depende da metodologia utilizada na coleta do dado.^2^

A análise de uma coorte do banco de dados do estudo REPLICCAR II, com 1387 pacientes submetidos a cirurgia de revascularização miocárdica (CRM) em 5 centros em São Paulo revelou que fatores como menor índice de massa corporal, histórico de infarto do miocárdio, diabetes mellitus, insuficiência renal e um elevado escore *Society of Thoracic Surgeon* estiveram fortemente associados a um maior risco de readmissão por todas as causas em até 5 anos. A incidência cumulativa de readmissão foi de 27,69%, com tempo médio de retorno ao hospital de 2,4 anos após a cirurgia.^3^

Essa análise coloca em evidência preditores que influenciam diretamente esse desfecho, reforçando a necessidade de abordagens preventivas e modelos preditivos mais precisos para otimizar os resultados a longo prazo de pacientes submetidos à CRM. A ausência de alguns dados impossibilita uma análise mais minuciosa das causas de readmissão e possíveis intervenções a serem realizadas, sendo mencionadas apenas se foram readmissões eletivas ou de emergência sem terem sido descritos o diagnóstico e o tempo de duração das internações.

O estudo utilizou como metodologia de acompanhamento o contato telefônico. Mais da metade dos pacientes do número total do banco de dados (N=4045) não pôde ser contatado. Vieses de análise na perda desses pacientes podem ter ocorrido, pela possibilidade de que outros fatores de risco poderiam estar associados com o desfecho final. A readmissão hospitalar esteve associada a maior mortalidade, portanto esse dado levanta a possibilidade de que pacientes que possam ter evoluído a óbito e não puderam ser contatados tenham em algum momento sido readmitidos.

Um aspecto importante levantado pelo estudo foi justamente a necessidade de unificação de banco de dados de cirurgia cardíaca no Brasil. Um banco se está relacionado ao Sistema de Informação de Mortalidade disponível no Brasil, traz a informação real dos pacientes de uma análise que foram a óbito.^4^ Reforça-se, portanto, a importância do relacionamento dos centros de pesquisa com dados do Ministério da Saúde.

Bancos de dados nacionais representam realmente uma estratégia difícil de ser atingida na maioria das nações, porém exemplos como o da Dinamarca evidenciam o êxito na análise de patologias específicas. Já estão bem consolidados no país há mais de 40 anos; o DBCG ( *Danish Breast Cancer Group* ) foi o primeiro com propósito de pesquisa e o DLCR ( *Danish Lung Cancer Register* ) foi o primeiro que teve como foco primário a qualidade do cuidado. O *Danish Heart Regi* stry é um banco de dados nacional que coleta dados médicos e administrativos de pacientes que realizam procedimentos cardiológicos invasivos e cirúrgicos e é utilizado para fins de análise e planejamento de qualidade assistencial e de remuneração às instituições.^5 , 6^ Trata-se da transição de um modelo baseado em volume para um focado na geração de valor.

Uma avaliação dos centros participantes em um estudo que avalia a qualidade assistencial em cirurgia cardíaca é fundamental na análise dos resultados. Já foi demonstrado, inclusive, que a mortalidade cirúrgica de diferentes cirurgiões cardíacos variou de acordo com a instituição em que as cirurgias foram realizadas.^7^

Além dos dados analisados no estudo, existem também estudos que demonstraram significância estatística quanto à readmissão após cirurgia cardíaca quando foram analisados dados socioeconômicos e tempo de internação durante a cirurgia cardíaca, sendo inequidades no acesso à cirurgia e tempo mais prolongado de internação fatores que aumentam a chance de admissão a médio e longo prazo.^8 , 9^

Mecanismos de medição são realmente essenciais para uma cultura de segurança e qualidade assistencial, e demonstram a existência de um caminho a ser seguido. Após a coleta de dados, é preciso realizar a análise deles que deve ser seguida por elaboração de um plano de ação de melhorias e implementação do mesmo visando a melhoria no cuidado aos pacientes submetidos à CRM.
