# Supplementary material for: Preditores de Readmissão Hospitalar Após Cirurgia de Revascularização Miocárdica – Reflexões e Perspectivas
Source: Arq Bras Cardiol. 2025 Apr 7;122(2):e20250133. [Article in Portuguese] doi: 10.36660/abc.20250133 (PMC12040383; doi:10.36660/abc.20250133)
Supplement: Translation

# Translation: Predictors of Hospital Readmission After Coronary Artery Bypass Grafting – Reflections and Perspectives

**DOI:** 10.36660/abc.20250133i

**Authors:** Aurora Felice Castro Issa

**Affiliations:** 1 Rio de Janeiro RJ Brasil Instituto Nacional de Cardiologia, Rio de Janeiro, RJ – Brasil; 2 Rio de Janeiro RJ Brasil IDOMED, Rio de Janeiro, RJ - Brasil

**Keywords:** Patient Readmission, General Surgery, Myocardial Revascularization

Aspects such as the high cost of health care, the overload of health professionals, and the aging population with the consequent increase in chronic diseases highlight the need for attention to quality in highly complex cardiovascular care, especially in cardiac surgery.^1^In this scenario, quality indicators are an extremely important instrument in the evaluation of care.

There are several quality indicators currently available, and one of them is the hospital readmission rate, which can be seen as an outcome often used as secondary and, despite being quite promising, depends on the methodology used to collect the data.^2^

Analysis of a cohort from the REPLICCAR II study database of 1,387 patients undergoing coronary artery bypass grafting (CABG) in 5 centers in São Paulo revealed that factors such as lower body mass index, history of myocardial infarction, diabetes mellitus, renal failure, and a high Society of Thoracic Surgeon score were strongly associated with a higher risk of all-cause readmission within 5 years. The cumulative incidence of readmission was 27.69%, with a mean time to return to the hospital of 2.4 years after surgery.^3^

This analysis highlights predictors that directly influence this outcome, reinforcing the need for preventive approaches and more accurate predictive models to optimize long-term results for patients undergoing CABG. The absence of some data makes it impossible to conduct a more detailed analysis of the causes of readmission and possible interventions, with only elective or emergency readmissions mentioned without describing the diagnosis and length of hospitalization.

The study used telephone contact as a follow-up methodology. More than half of the patients in the total number of patients in the database (N=4045) could not be contacted. Analysis biases in the loss of these patients may have occurred due to the possibility that other risk factors could be associated with the outcome. Hospital readmission was associated with higher mortality; therefore, this data raises the possibility that patients who may have died and could not be contacted were readmitted at some point.

An important aspect raised by the study was precisely the need to unify the cardiac surgery database in Brazil. A database that is linked to the Mortality Information System available in Brazil provides real information on patients who died from an analysis.^4^This reinforces the importance of the relationship between research centers and data from the Ministry of Health.

National databases are indeed a difficult strategy to achieve in most nations, but examples such as Denmark demonstrate success in analyzing specific pathologies. They have been well established in the country for over 40 years; the DBCG (Danish Breast Cancer Group) was the first with a research purpose and the DLCR (Danish Lung Cancer Register) was the first with a primary focus on quality of care. The Danish Heart Registry is a national database that collects medical and administrative data on patients undergoing invasive and surgical cardiological procedures and is used for the purposes of analyzing and planning care quality and remuneration for institutions.^5 , 6^This is the transition from a model based on volume to one focused on value generation.

An assessment of the centers participating in a study that assesses the quality of care in cardiac surgery is essential in the analysis of the results. It has even been demonstrated that the surgical mortality of different cardiac surgeons varied according to the institution where the surgeries were performed.^7^

In addition to the data analyzed in the study, some studies demonstrated statistical significance regarding readmission after cardiac surgery when socioeconomic data and length of hospital stay during cardiac surgery were analyzed, with inequities in access to surgery and longer hospital stays being factors that increase the chance of admission in the medium and long term.^8 , 9^

Measurement mechanisms are truly essential for a culture of safety and quality of care and demonstrate the existence of a path to be followed. After collecting data, it is necessary to analyze it, which should be followed by the development of an action plan for improvements and its implementation, aiming at improving the care of patients undergoing CABG.
